# A Systematic Review and Meta-analysis of Randomized Controlled Trials on the Effect of Transcranial Magnetic Stimulation on Tinnitus Management

**DOI:** 10.5195/cajgh.2020.356

**Published:** 2020-03-31

**Authors:** Salma Galal, Naema Ismail, Ghada Niel

**Affiliations:** 1 Community and Industrial Medicine Department, Faculty of Medicine; 2 Audiology Department, Faculty of Medicine, Al-Azhar University, Cairo, Egypt; 3 Audiology Department, Mansoura International Hospital, Mansoura, Egypt

**Keywords:** Tinnitus, TMS, Transcranial Magnetic Stimulation, Magnetic Field Therapy

## Abstract

**Introduction:**

Tinnitus occurs in 10–15% of the world's population. It may lead to hearing loss, depression, and suicidal tendencies, as well as reduced quality of life. The aim of this study was to assess whether Transcranial Magnetic Stimulation (TMS) effectively reduces tinnitus handicapping after six months or more of follow-up.

**Methods:**

A systematic review of randomized controlled trials with follow-up of six months was undertaken. The review took place through searching Medline, Science Direct, and Google Scholar databases using the keywords “tinnitus” and “Transcranial Magnetic Stimulation” and limiting the search results to randomized controlled trials (RCTs) conducted on adults (19 years and older) published between 2005–2015. Meta-analysis was performed on the similarly designed studies.

**Results:**

Five RCTs with six month follow-up were found conforming to the inclusion criteria. In total, there were 119 patients in the TMS arms and 115 in the placebo arms. However, designs were different between the studies and were therefore not all comparable. Different parameters were used to measure the severity of tinnitus and depression scores. Tinnitus handicapped inventory (THI) was the common measured outcome parameter used in all studies. THI score decreased after the TMS in four studies. Meta-analysis was performed on three similarly designed RCTs with the overall effect being insignificant.

**Conclusions:**

TMS reduced the THI score and decreased the severity of tinnitus in 45% of patients and lead to a complete recovery in 32% of cases in one study. However, the meta-analysis demonstrated lack of significant effect of TMS on tinnitus management.

Tinnitus is the perception of sound in the ear or in the head without any external acoustic stimulation. Numerous hypotheses have been developed for the pathophysiology of tinnitus. It has been suggested that tinnitus may arise from any abnormality of the neural pathway from the cochlear neural axis to the auditory cortex.^1^ The pathophysiological theory implies that the central nervous system is the source or “generator” of tinnitus.^2^ Tinnitus is often a feature of ear disease and is usually associated with hearing loss, but it may also occur in patients with normal hearing.^3^ Many cases of tinnitus have no identifiable cause. Environmental exposure to recreational, urban, and occupational noise or ototoxic drugs can develop tinnitus.^4^ Explosion or firing can cause damage to the peripheral auditory organs, which in turn causes the activation of neural plasticity and leads to tinnitus.^5^

In 39 studies done in Belgium, Italy, Denmark, Finland, Norway, Sweden, UK, Scotland, USA, Japan, China, South Korea, Australia, Egypt, Nigeria, and Brazil, the prevalence of tinnitus ranges from 5.1% to 42.7% and is higher in males than in females.^6^ The National Health Interview Survey found that, within the US population, 11.2% of adults and 7.5% of adolescents suffer from tinnitus; tinnitus prevalence increases with age.^7^,^8^ In 1–2% of people who have tinnitus, tinnitus symptoms seriously reduce the quality of life, resulting in social isolation, depression, and even suicidal tendencies.^5^

In chronic cases, a variety of treatment approaches are available, including pharmacological treatment, complementary and alternative medicine therapies, sound treatment/associated technologies, psychological/behavioral treatment, and cochlear implants. There is no pharmacological treatment for tinnitus with long-term effect.^9^ Talk therapy and sound therapy with little support of medication are the primary treatment in developed countries.^10^,^11^ There is little evidence on tinnitus management forms using Chinese, alternative or complementary medicine. These therapy methods include Ginkgo biloba, melatonin, zinc, diet modification, hyperbaric oxygen, temporo-mandibular joint therapy, and acupuncture, among others.^12^

Tinnitus treatment can be reached by interrupting the abnormal activity and neuro-modulation.^13^ Repetitive magnetic fields generated by repetitive Transcranial Magnetic Stimulation (rTMS) can reduce neural overactivity in cortical areas and can potentially alleviate tinnitus.^14^ It is a non-invasive procedure.^15^ Meng et al. review on tinnitus management with TMS suggests addressing its long-term effectiveness.^9^ Recent and ongoing research studies have attempted to assess whether rTMS could be an effective tinnitus treatment for a longer duration. Therefore, the aim of this study was reviewing RCTs that addressed the effect of TMS on tinnitus after at least six months.

## Methods

### Search strategy

Electronic searches on the Medline (PubMed), Science Direct, and GoogleScholar databases were carried out in February 2016. English language articles published between 2005 and 2015 were selected. Cochrane Library was searched for systematic reviews on the topic. The search keywords used were either “unilateral or bilateral tinnitus”, “Trans-cranial Magnetic Stimulation”, “TMS”, “TMS treatment”, “repetitive TMS” and “rTMS”. Only RCTs with adults at least 19 years old and at least six months follow-up were included. The authors independently searched the sites, reviewed the titles, abstracts, and keywords, and agreed on the studies included in the review. The decision for a final inclusion of the studies was made after reviewing the full articles. The authors resolved differences by discussing them together. The libraries of the Faculties of Medicine in some Egyptian Universities were searched on the same topic by another author. No thesis was found on the systematic review of rTMS for tinnitus treatment.

### Study inclusion and exclusion criteria

Any RCT using rTMS treatment (low/high frequency) with at least six months of follow-up was considered eligible. Studies with children under the age of 19 or adults with total hearing loss. Studies with combined therapy, where rTMS treatment was used in conjunction with pharmacological therapy, diet modification, psychotherapy, hearing aids, or any metal appliances were also excluded. Different tools are used in RCTs to measure the severity of tinnitus. The authors tried to find one common primary or secondary tool for measuring severity, which was ultimately determined to be tinnitus handicapped inventory (THI).

### Data extraction

General information on publication, authors, article title, journal title, and publication year was extracted. The design of the trial was assessed in regard to trial arms, sample size, randomization process, allocation method, blinding of information, and statistical methods. The total number of intervention and comparison groups of participants was registered with baseline characteristics, age, gender, inclusion and exclusion criteria. The intervention with TMS pulse, stimulus frequency, and dropouts were reviewed. Primary and secondary outcomes such as THI and depression or anxiety tests at baseline, at the end of the treatment and at follow-up were assessed. The number and type of adverse events were also extracted. The conclusion was considered. The review authors assessed the risk of bias in the included studies. The authors collected and extracted data from each RCT study included and authors of the primary studies were contacted to clarify any questions about the data.

### Data synthesis

A descriptive data synthesis was done according to the reporting of the studies. In addition, meta-analysis of three studies with similar design was carried out in Review Manager 5.

**Figure 1.**
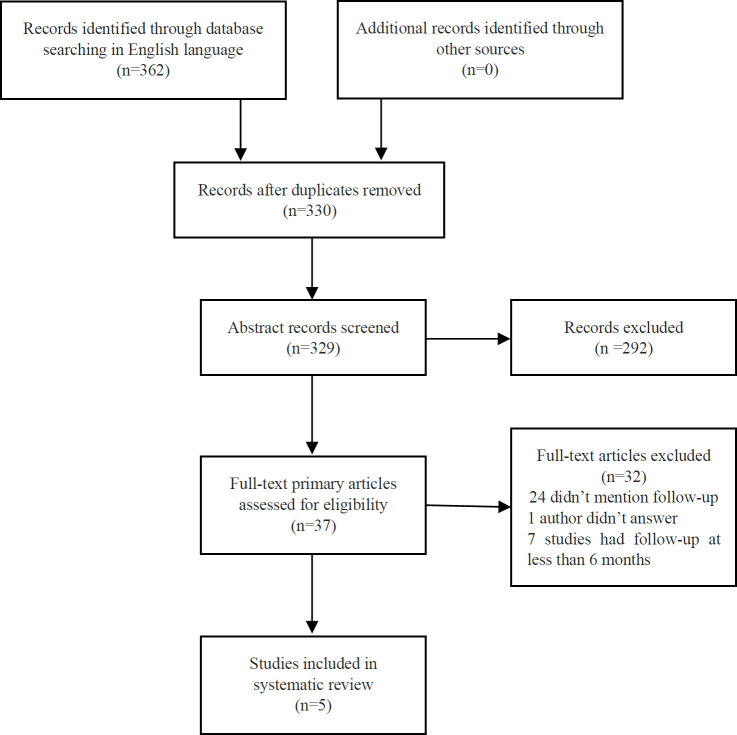
Flowchart of the review: RCTs on tinnitus management with rTMS

## Results

The electronic search using the keywords for studies from 2005–2015 identified 362 articles: 240 from Google Scholar, 46 from Medline, and 76 from Science Direct. After removing duplicates, the authors screened 329 studies—292 by abstract and 37 by full text—according to the criteria of the search; 292 were excluded, 24 studies did not mention the follow-up, and seven studies had follow-ups less than six months. Landgrebe et al. study had to be excluded, as the corresponding author did not respond to the authors’ questions.^21^ The five studies eligible for inclusion were: Andres et al., Hoekstra et al., Khedr et al., Kim et al., and Marcondes et al.^16^^–^^20^

### Five included studies

All five studies included in this review were randomized controlled double-blind trials from Czech Republic, Netherlands, Egypt, Korea, and Brazil investigating the efficacy of rTMS for at least six months post treatment. Khedr et al. followed up monthly for 10 months.^18^ Studies were published in 2010–2014. All studies used low-frequency 1-Hz rTMS in 2-trial arms except Khedr et al. who had 4-trial arms assessing 1-Hz rTMS versus 25-Hz rTMS and ipsilateral rTMS against contralateral.^18^ Three studies compared rTMS with sham, unlike Kim et al. and Khedr et al.^18^,^19^ All studies enrolled 19 to 62 chronic tinnitus patients with different conditions. ^17^ They were assigned randomly to the trial arms. Diverse primary and secondary tools were used to measure the outcomes. The tinnitus handicapped inventory (THI) and the visual analogue rating scores (VAS) were used to measure outcomes in all studies alongside diverse other tools at baseline, during follow-up, and after six months.

### Analysis of studies

Random allocation was described in all studies except for Marcondes et al. study.^20^ The blinding process was explained in all trials except for in Kim et al.^19^ All studies had 3.8% (low risk) to 19.6% (high risk) dropouts except for Khedr et al. with no dropouts.^18^ Reasons for dropping out given by Kim et al. were four patients received additional treatment during follow up and one patient had severe headaches.^19^ During the rTMS treatment no serious side-effects were reported. Nine patients from all studies experienced headache as adverse effects and only sporadic dizziness, pain at the site of stimulation, and sleep pattern changes.

Diverse scales were applied to measure the primary and secondary outcomes, however, tinnitus handicapped inventory (THI) was used in all studies. Only two studies had scales for secondary outcome.^17^,^18^ The measurements were taken at baseline, after rTMS treatment or placebo, 2–10 times during follow-up and six months after the intervention. Only one study measured them after 10 months. Andres et al. found significant reduction of the total score of basic scales that measure tinnitus severity.^16^ Hoekstra et al. pointed out that tinnitus was unchanged. ^17^ Khedr et al. revealed that 32.25% of all patients recovered completely from tinnitus and 27.4% improved in having tinnitus only at night before sleeping.^18^ Kim et al reported improvement in 46.7% of the ipsilateral group and 51.6% of the contralateral group.^19^ For Marcondes et al., 40% had a significant reduction of tinnitus severity after five days and for one to six months after treatment of active rTMS.^20^ Overall, more than 45% of patients experienced improvement. Three of the studies assessing depression and anxiety with different scales did not find any differences between the groups during follow-up.^16^^–^^18^ Khedr et al. used VAS for loudness, awareness, and annoyance level of symptoms. After 10 months follow-up, the contralateral group showed more improvement regarding the annoyance level than the ipsilateral group.^18^ In the study of Kim et al., the annoyance level did not show a significant difference.^19^

Although the comparison between high and low rTMS and ipsilateral and contralateral is of importance, the aim of our study implies the comparison of rTMS versus ‘sham’ which was applied in three studies.^16^,^17^,^20^ THI was the common scale used for the comparison of outcomes at baseline, during follow-up, and six months after the intervention. There was improvement in the THI scores in the rTMS group in the RCTs of Andres et al. and Marcondes et al., but not in Hoesksta et al.^16^,^17^,^20^ No significant differences were found between rTMS and the sham group in all three studies at baseline, during follow-up, or six months after the intervention, except in the study of Marcondes et al. directly after rTMS.^20^

**Figure 2.**
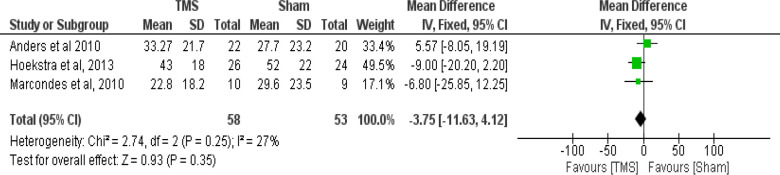
Forrest-plot showing the mean THI scores in rTMS versus Sham (1–4 weeks post-intervention) in the three studies

**Figure 3.**
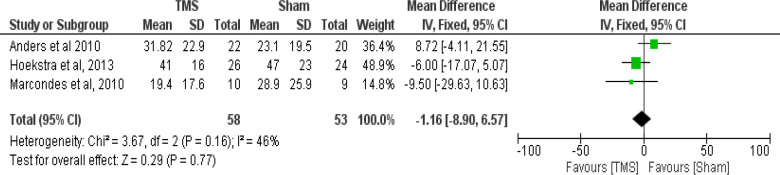
Forrest-plot showing the mean THI scores in rTMS versus Sham (6 months post-intervention) in the three studies

Meta-analysis of the three studies with similar design was performed. Two separate comparisons between the outcomes of the THI scores in rTMS and sham group were set using data derived from the three studies. The first comparison at 1–4 weeks post-intervention favored the rTMS intervention over the sham but not to a statistically significant level (Test of overall effect: Z = 0.29, P = 0.77; Fig. 2). The second comparison at six months post-intervention also favored the rTMS intervention over the sham but not to a statistically significant level (Test of overall effect: Z = 0.93, P = 0.35; Fig. 3).

## Discussion

According to this systematic review, rTMS reduced the severity of tinnitus in four RCTs in over 45% of the patients for a duration of six months following the intervention. Around one third of patients in one study were completely recovered from tinnitus.^18^ Only one study did not find any changes.^17^ The outcome differences of the THI scores of the trials is due to diverse inclusion criteria of patients or technical application of rTMS. Two studies measuring depression and anxiety did not find any changes after rTMS application despite reduction of tinnitus. It is likely that depression and anxiety take longer to improve, which explains the accompanying use of talk therapy in some regimens.^10^,^11^

As the primary aim of our review was to compare rTMS with ‘sham’, only three studies matched.^16^,^17^,^20^ Andres et al. reported significant reduction of the total score of basic scales that measure tinnitus severity, even for patients with a mean duration of nine years not responding to pharmacological treatment.^16^ For 40% of patients exposed to rTMS in the Marcondes et al. trial, the tinnitus severity decreased as measured with the THI.^20^ In contrast, the study of Hoekstra et al. indicated no changes,^17^ likely due to this study including non-fluctuating tinnitus patients while the other two trials mentioned just unilateral and bilateral tinnitus patients.

Some other inclusion criteria such as hearing loss can have an effect on the outcome. Marcondes et al.^20^ reported a positive effect of rTMS on subjects with normal hearing. Hearing loss might influence the effect of rTMS. The trial of Khedret al.^18^ reported that hearing impairment might exacerbate the plastic changes in neural function causing tinnitus, and that decreases the effect of rTMS. This is in agreement with the study of Kleinjung et al. and Smith et al. reporting on the negative influence of hearing loss on the efficacy of rTMS.^13^,^22^ In contrast, Lehner et al. did not find a relationship between hearing loss and rTMS efficacy.^23^ Andres et al. included only normal hearing patients.^16^ The studies of Hoekstra et al. and Kim et al. did not report on this issue in their results, although they both included patients with impaired hearing.^17^,^19^

In addition, all studies included chronic tinnitus patients. Duration of tinnitus is another one of the inclusion criteria that can affect the outcome. Tinnitus duration should be considered when explaining the different outcomes between the five included studies. Khedr et al.'s^18^ trial showed that there was a significant correlation between the duration of symptoms and change in THI (at baseline and 10 months after). This is substantiated through other studies that found patients who had the shortest history of tinnitus tended to respond the best to rTMS therapy,^13^,^24^^–^^26^ though other studies did not find this effect.^22^, ^23^ Andres et al. stated that their trial lowered the severity of tinnitus even in chronic patients who had it for nine years.^16^ The other three studies did not mention the effect of tinnitus duration on the outcome.^17^,^19^,^20^

Another clinical implication of our review suggests that low-frequency rTMS, ipsi- or contralateral positioning of the coil on the temporo-parietal cortex or auditory cortex reduces the severity of tinnitus. The auditory cortex is thought to play an important role in tinnitus, but there is strong evidence that the auditory cortex together with the limbic system, prefrontal and parietal cortex determines tinnitus distress.^27^^–^^30^ The parietal cortex and its connections to the auditory cortex could be involved in tinnitus through the mediating effect that the parietal cortex has on auditory attention.^31^, ^32^ Repetitive TMS of these areas could therefore decrease a patient's reaction to tinnitus, leading to a reduction in the perception of tinnitus. Another study reported that a combination of temporal and prefrontal stimulation showed a significant effect on tinnitus.^13^ Repetitive TMS works by interfering with baseline activity in the cortex and decreases tinnitus. This opinion is confirmed by Smith et al. who found greater response of the contralateral stimulation using low-frequency rTMS.^22^ In contrast Kim et al.'s trial found no significant difference between ipsilateral and contralateral stimulation, and tinnitus was reduced in half of the patients regardless of the side of stimulation.^19^ Hoekstra et al. found no effect of bilateral stimulation of the auditory cortex.^17^ Marcondes et al. did not mention this point.^20^ The use of low-frequency rTMS was applied by the five trials, which is contrary to Meng et al. who found “very limited support for the use of low-frequency rTMS for the treatment of patients with tinnitus” after four months of follow-up.^9^

The duration of rTMS is another factor that might influence its effect. In Andres et al. trial and Khedr et al. the patients were treated for two weeks.^16^,^18^ In Marcondes et al., Hoekstra et al., and Kim et al., the patients were treated for one week.^17^,^19^,^20^ It is reported that results may be better after a longer duration of treatment over two weeks.^33^

Meta-analysis was not applied to all the RCT studies as they differed in their design (Table 1). Kim et al. used ipsilateral versus contralateral.^19^ Khedr et al. had four trial arms comparing between high- and low-frequency and ipsilateral versus contralateral.^18^ Three RCTs abided to the primary aim of our study, using rTMS versus sham in the trial arms: Andres et al., Hoekstra et al., and Marcondes et al.^16^,^17^,^20^ The tinnitus handicapped inventory (THI) was used as the measurement for tinnitus severity by all studies. The meta-analysis was performed on those RCTs with comparable design.^16^,^17^,^20^ The rTMS intervention was favored, but without statistically significant effect. More than three identified RCTs for the meta-analysis would have given stronger evidence. The limitations of this review were lack of funding, differences in protocols of the studies, its performance on limited database, and using only articles published in English.

**Table 1.** Patients with tinnitus with rTMS intervention for randomized controlled studies (RCT) with at least 6 months of follow-up

Authors	Date published	Study design	Number of trial arms	Patients’ Number	Inclusion criteria	Duration of intervention	Site	Primary Outcome	Secondary Outcome	Time of measurement	Comments
Andres et al.^18^	2010	randomized, prospective, placebo-controlled	1- Hz rTMS Sham	22 20	chronic uni- or bilateral tinnitus patients of ~9 years duration Normal hearing Right handed	2 weeks	Czech Republic Psychiatry Otorhinolaryngology, Neurology, Radiology Charles University in Prague	TQ^*^ modified THI^**^ VAS1^***^ VAS2 Goebel & Hiller tinnitus questionnaire		-before start -after 2 – 6 – 14 – 26 weeks	significant reduction of the total score of basic scales that measure tinnitus severity. reduction was displayed in figures
Hoekstra et al.^19^	2013	RCT block design per group of 8, double-blind placebo-controlled	1 -Hz rTMS Placebo	26 24	chronic non-fluctuating tinnitus of 8 months with some hearing loss	5 consecutive days	Netherlands Otorhinolaryngology University Medical Center Utrecht &Brain Center	TQ	THI -VAS STAI^****^ Beck Depression Inventory	-before start-after last session-after 1 week -after 1-3-6 months	Tinnitus unchanged. 25% improvement on the TQ.
Khedr et al.^20^	2010	RCT randomized to four groups	*1- Hz rTMS*: Ipsilateral Contralateral *25 -Hz rTMS*: Ipsilateral Contralateral	15 16 15 16	Right or left ear tinnitus Normal hearing & some hearing loss	2 weeks daily	Egypt NeuroPsychiatry, Audiology Assiut University Hospital	THI VAS	RI and Hamilton ratings of depression and anxiety	-before start-after last session-monthly interval for 10 months	32.25% of all patients recovered completely from tinnitus. 27.4% improved to the point where they only had tinnitus at night before sleeping. In the contralateral group 64.5% improved in comparison to 29% in the ipsilateral group. No different effect of 1Hz or 25 Hz frequency.
Kim et al.^21^	2014	RCT patients were assigned randomly to the ipsilateral orcontralateral	1- Hz rTMS : Ipsilateral Contralateral	30 31	tinnitus localized to poor ear asymmetric hearing impairment at least 6months & treated for at least 2 months	for 5 days	Korea Dept Otorhinolaryngology, Research Institute of Rehabilitation	THI VAS:- loudness awareness annoyance		-before start-after last session-after 1-3-6 months	46.7% of patients having ipsilateral stimulation and 51.6% of contralateral showed improvement
Marcondes et al.^22^	2010	RCT Randomized double-blind controlled	1 -Hz rTMS Placebo	10 9	uni- or bilateral tinnitus of 3 months duration, normal hearing	5 consecutive days	Brazil Dept Otolaryngology, Radiology, Psychiatry	THI VAS SPECT^*****^		-before start-after 7- 14- 21days- monthly interval for 6 months	major changes in the physical & catastrophic domain 40% had a significant reduction of tinnitus severity after 5 days and 1, 6 months after treatment of active rTMS

* TQ = tinnitus questionnaire

** THI = tinnitus handicapped inventory

*** VAS = visual analogue rating scores

**** STAI = State-Trait Anxiety Inventory

***** SPECT = Single photon emission computed tomography

**Table 2.** Mean tinnitus handicapped inventory (THI) in 3 studies with similar design at baseline, directly after rTMS and after 6 months

			Baseline	THI After rTMS	After 6 months
Studies	Number of patients in trial arm	Mean age (years)		Mean THI ± SD	
Andres et al. (2010)	rTMS 22	48.09	37.09±21.7	31.82±22.9 (2 weeks)	33.27±21.6
	Sham 20	50.05	26.5±20.4	23.1±19.5	27.7±23.2
Hoekstra et al. (2013)	rTMS 26	50	45 ±21	41 ±16 (1 week)	43 ±18
	Sham 24	55	44± 22	47 ±23	52 ± 22
Marcondes et al. (2010)	rTMS 10	–	29.8 ± 22.8	19.4 ± 17.6* (1 month)	22.8 ± 18.2
	Sham 9	–	28.9 ± 23.8	28.9 ± 25.9	29.6 ± 23.5

– patients were more than18 years of age

* p = 0.047 one sided (significant)

Tinnitus handicapped inventory (THI) scores indicate that rTMS has a role in decreasing the severity of tinnitus. It sustained the improvement and reduced handicapping for the duration of six months in three RCTs or, as is the case in one trial, even 10 months. Four studies reported reduction in tinnitus severity after rTMS in over 45% of patients even after six months follow-up.^16^,^18^^–^^20^ One of the four studies had one third of patients completely recovered from tinnitus.^18^ Only one study found rTMS not effective on any outcome parameter.^17^

Although the meta-analysis of the three studies with similar design of rTMS and sham favored rTMS intervention, the overall statistical effect showed no significant difference between the groups, regarding the tinnitus handicapped inventory (THI) scores. Given the scarce number of RCTs between 2005 and 2015, more studies in multi-centers with the same protocol of design, inclusion/exclusion criteria, technological procedure, and outcome measurements will provide stronger evidence. Follow-up in future studies should preferably be longer than six months to accrue stronger evidence.
